# Scientometric review of research on Neglected Tropical Diseases: a 31-year perspective from the Journal of the Brazilian Society of Tropical Medicine

**DOI:** 10.1590/0037-8682-0403-2022

**Published:** 2023-01-23

**Authors:** Anderson Fuentes Ferreira, Jorg Heukelbach, Carlos Henrique Nery Costa, Eliana Amorim de Souza, Adjoane Maurício Silva Maciel, Dalmo Correia, Alberto Novaes Ramos

**Affiliations:** 1 Universidade Federal do Ceará, Faculdade de Medicina, Programa de Pós-graduação em Saúde Pública, Fortaleza, CE, Brasil.; 2 Universidade Federal do Piauí, Departamento de Medicina Comunitária, Teresina, PI, Brasil.; 3 Centro de Inteligência em Agravos Tropicais Emergentes e Negligenciados, Teresina, PI, Brasil.; 4 Universidade Federal da Bahia, Instituto Multidisciplinar de Saúde, Campus Anísio Teixeira, Vitória da Conquista, BA, Brasil.; 5 Universidade Federal do Triângulo Mineiro, Programa de Pós-graduação em Medicina Tropical e Infectologia, Uberaba, MG, Brasil.; 6 Universidade Federal de Sergipe, Programa de Pós-graduação em Ciências da Saúde, Aracaju, SE, Brasil.; 7 Universidade Federal do Ceará, Faculdade de Medicina, Departamento de Saúde Comunitária, Fortaleza, CE, Brasil.

**Keywords:** Neglected Tropical Diseases, Citation Databases, Scientometrics, Tropical Medicine

## Abstract

**Background:**

To analyze the temporal evolution of research on Neglected Tropical Diseases (NTDs) published by the *Journal of the Brazilian Society of Tropical Medicine* (*JBSTM*).

**Methods:**

We performed an analysis of the scientific production in *JBSTM* on NTDs using an advanced search, which included authors’ descriptors, title, and abstract, and by combining specific terms for each NTDs from 1991 to 2021. Data related to authors, countries of origin, institutions, and descriptors, were evaluated and analyzed over time. Bibliographic networks were constructed using VOSviewer 1.6.16.

**Results:**

The *JBSTM* published 4,268 scientific papers during this period. Of these 1,849 (43.3%) were related to NTDs. The number of publications on NTDs increased by approximately 2.4-fold, from 352 (total 724) during 1991-2000 to 841 (total 2,128) during 2011-2021, despite the proportional reduction (48.6% *versus* 39.5%). The most common singular NTDs subject of publications included Chagas disease (31.4%; 581/1,849), leishmaniasis (25.5%, 411/1,849), dengue (9.4%, 174/1,849), schistosomiasis (9.0%; 166/1,849), and leprosy (6.5%, 120/1,849), with authorship mostly from Brazil’s South and Southeast regions.

**Conclusions:**

Despite the proportional reduction in publications, *JBSTM* remains an important vehicle for disseminating research on NTDs during this period. There is a need to strengthen the research and subsequent publications on specific NTDs. Institutions working and publishing on NTDs in the country were concentrated in the South and Southeast regions, requiring additional investments in institutions in other regions of the country.

## INTRODUCTION

Neglected tropical diseases (NTDs) cause high morbidity and mortality and are strongly associated with poverty and inequality. They affect populations with limited access to health services; have varied biological and transmission characteristics; and can be caused by fungi, viruses, bacteria, protozoa, arthropods, snakes, and helminths^1^ ^,^ ^2^. NTDs are present in more than 149 countries and have an annual financial impact on the world economy of billions of dollars^3^. In addition, approximately two billion people are at risk of developing one or more NTDs^3^ ^,^ ^4^.

The World Health Organization (WHO) list of NTDs has been amended over the previous decades^3^ ^,^ ^5^ ^,^ ^6^. In 2019, 20 diseases and/or groups of diseases were considered: Buruli ulcer, Chagas disease (American trypanosomiasis), dengue, chikungunya, dracunculiasis (guinea-worm disease), echinococcosis, foodborne trematode infections, human African trypanosomiasis (sleeping sickness), leishmaniasis, leprosy (Hansen’s disease), lymphatic filariasis (elephantiasis), mycetoma, chromoblastomycosis, and other deep mycoses; onchocerciasis (river blindness); rabies, scabies, and other ectoparasitoses; schistosomiasis (bilharzia); soil-transmitted helminthiases; snakebite envenoming; taeniasis and cysticercosis; trachoma; and yaws (endemic treponematoses)^3^. The definition of this group of diseases has yet to be unanimously decided upon, and it is up to local governments to prioritize investments and strategic actions for their control^3^ ^,^ ^7^.

Worldwide, five NTDs are the most commonly studied: dengue, soil-transmitted helminthiases, leishmaniasis, Chagas disease, and schistosomiasis. Among these diseases, dengue is the fastest-growing in terms of the number of publications associated with people living in resource-poor communities in urban centers in developing countries, in addition to epidemic areas in Southeast Asia and Brazil^8^.

As in other countries, the classification of NTDs in Brazil has changed over time as diseases are included and removed. In 2005, the Ministry of Health of Brazil research and development program considered the addition of six diseases to the list of epidemic/endemic NTDs: dengue, malaria, tuberculosis, Chagas disease, leishmaniasis, and leprosy. In 2008, schistosomiasis was added. The report “*Health Brazil 2017: an analysis of the health situation and the challenges for the achievement of the Sustainable Development Goals*” under Chapter 5 (“*Neglected diseases in Brazil: vulnerability and challenges*”) considered Chagas disease, schistosomiasis mansoni, leprosy, lymphatic filariasis, tegumentary leishmaniasis, visceral leishmaniasis, onchocerciasis, human rabies, and trachoma to be NTDs^7^ ^,^ ^9^. The WHO list includes NTDs present in Brazil, Latin America, and other African and Asian continents^3^.

Each of these diseases has specific characteristics in various Brazilian epidemiological contexts; some are in the different stages of control^10^. Considering the current pandemic caused by the new coronavirus (SARS-CoV-2) and its potential impact, the future epidemiological scenario for NTDs is uncertain owing to the weakening of control actions, as well as limited access to health care, diagnosis, and treatment for affected people. There are delays in manufacturing, shipment, transport, and delivery of drugs; reassignment of personnel; and diversion of financial resources. Currently, areas negatively impacted by COVID-19 primarily include community interventions; early diagnosis; access to treatment and care in the health services network; monitoring and evaluation activities; reallocation of financial resources to other “*priority*” actions; and relocation of teams for surveillance, care, and control of NTDs to support actions to combat the virus^11^.

The official journal of the Brazilian Society of Tropical Medicine (BSTM), the *Journal of the Brazilian Society of Tropical Medicine* (*JBSTM*), presents primarily in the context of Brazilian and Latin American spaces and follows a multidisciplinary perspective. The papers can be assessed openly, and submissions are published 100% free of charge. The journal publishes original research on NTDs, preventive medicine, public health, infectious diseases, and related subjects. *JBSTM* was founded in 1962 as a channel for scientific dissemination. The first issue was released in 1967, with five articles on schistosomiasis^12^. Here, we present a scientometric review of NTDs in the *JBSTM* from 1991 to 2021.

## METHODS

### Type of study

The current study is a scientometric review of NTDs published by *JBSTM* and indexed in the Scopus® database accessed through the Federated Academic Community of the Coordination for the Improvement of Higher Education Personnel (in portuguese: *Comunidade Acadêmica Federada da Coordenação de Aperfeiçoamento de Pessoal de Nível Superior* [CAFe-CAPES]) (https://www.scopus.com/home.uri). All types of scientific productions on NTDs published by *JBSTM* were included, independent of the country of origin and publication: original articles, editorials, review articles, short communications, case reports, technical reports, images of infectious diseases, letters, supplements, and obituaries.

### Source and organization of data

Database searches (deadline: July 27, 2022) were completed using specific criteria and the advanced search function by applying the author's descriptors, title, and abstract, combining specific terms for each NTD (Table 1). The NTDs were selected based on a group of 20 diseases defined by WHO^2^. All publications from January 1991 to December 2021 were considered and screened. Data on all authors, country of origin, institutions, and health descriptors were extracted and analyzed, and the number and proportion of scientific production over time was evaluated. All authors (with their country of origin and affiliation) included in the scientific publications were considered.

**TABLE 1:** Neglected Tropical Diseases (NTDs) and Search Terms used.

NTDs	Search Terms
Buruli ulcer	Buruli Ulcer; *Mycobacterium ulcerans*
Chagas disease	Chagas disease; *Trypanosoma cruzi*
Chromomycosis	Chromomycosis; *Phialophora*; *Rhinocladiella*; *Exophiala*; *Fonsecaea pedrosoi*; *Cladophialophora carrionii*
Taeniasis/cysticercosis	Cysticercosis; Taeniasis; *Taenia solium*; *Taenia saginata*
Dengue	Dengue; DENV; Flavivirus*
Chikungunya	Chikungunya Fever; Chikungunya virus; CHIKV
Echinocococosis/Hydatidosis	Echinococcosis; *Echinococcus granulosus*; *Echinococcus multilocularis*
Fascioliasis	Fascioliasis; *Fasciola gigantica*; *Fasciola hepatica*
Leishmaniasis	Leishmaniasis; *Leishmania donovani*; *Leishmania chagasi*; *Leishmania infantum*; *Leishmania major*; *Leishmania tropica*; *Leishmania braziliensis*; *Leishmania mexicana*; Leishmania*
Leprosy	Leprosy; *Mycobacterium leprae*
Elephantiasis; Elephantiasis, Filarial	Elephantiasis; *Elephantiasis*; Filarial; *Wuchereria bancrofti*; *Brugia malayi*; *Brugia timor*
Mycetoma	Mycetoma; *Nocardia brasiliensis*; *Nocardia asteroides*; *Nocardia otitidiscaviarum*; *Nocardia ninae*; *Gordonia terrae*; *Madurella mycetomatis*; *Fonsecaea pedrosoi*; *Acremonium falciforme*
Yaws	Yaws; *Treponema pallidum*
Onchocerciasis	Onchocerciasis; *Onchocerca volvulus*
Rabies	Rabies; Rabies virus
Schistosomiasis	Schistosomiasis; *Schistosoma haematobium*; *Schistosoma guineensis*; *Schistosoma intercalatum*; *Schistosomiasis japonica*; *Schistosoma mekongi*; *Schistosomiasis mansoni*
Trachoma	Trachoma; *Chlamydia trachomatis*
Ascariasis	Ascariasis; *Ascaris lumbricoides*; *Ascaris suum*
Trichuriasis	Trichuriasis; *Trichocephalus*; *Trichuris trichiura*
Ancylostomiasis	Ancylostomiasis; *Ancylostoma caninum*; *Necator americanus*
Dracunculiasis	Dracunculiasis; *Dracunculus medinensis*
Clonorchiasis	Clonorchiasis; *Clonorchis sinensis*
Paragonimiasis	Paragonimiasis; Paragonimus*
Opisthorchiasis	Opisthorchiasis; *Opisthorchis viverrini*; *Opisthorchis felineus*
Trypanosomiasis, African	Trypanosomiasis; African; *Trypanosoma brucei gambiense*; *Trypanosoma brucei rhodesiense*
Chromoblastomycosis	Chromoblastomycosis; *Fonsecaea pedrosoi*; *Phialophora verrucosa*; *Cladophialophora carrionii*; *Rhinocladiella aquaspersa*
Snake Bites	Snake Bites
Histoplasmosis	Histoplasmosis; *Histoplasma capsulatum*
Coccidioidomycosis	Coccidioidomycosis; *Coccidioides immitis*; *Coccidioides posadasii*
Paracoccidioidomycosis	Paracoccidioidomycosis; *Paracoccidioides brasiliensis*
Sporotrichosis	Sporotrichosis; *Sporothrix schenckii*
Cryptococcosis	Cryptococcosis; *Cryptococcus neoformans*; *Cryptococcus gattii*
Scabies	Scabies; *Sarcoptes scabiei*
Tungiasis	Tungiasis; *Tunga penetrans*
Cutaneous Larva Migrans	Cutaneous Larva Migrans; Visceral Larva Migrans; *Ancylostoma caninum*; *Ancylostoma brasiliensis*; *Strongyloides stercoralis*
Head Lice Infestations	Lice Infestations; *Pediculus humanus capitis*; Body lice; Phtiriase
Myiasis	Myiasis; *Cochliomyia hominivorax*; *Oestrus ovis*; *Wohlfahrtia magnifica*; *Chrysomya bezziana*; *Hypoderma bovis*; *Hypoderma lineatum*; *Cordylobia anthropophaga*; *Hypoderma tarandi*; *Calliphora vicina*; *Musca nebulo*; *Musca domestica*; *Lucilia sericata*

An author's country of origin and affiliated institutions were considered to be the first information reported by the author, as manuscripts were counted as a unit of analysis. The relationship analysis was carried out using the scientometric visualization software VOSviewer 1.6.16 (https://www.vosviewer.com/) based on the structuring of the bibliographic networks and the specificities of the references associated with each record, in addition to the author's descriptor data and the arrangement of the most frequent terms in the publications.

Data were analyzed for the complete period and stratified into four periods (1991-2000, 2001-2010, 2011-2021, and 1991-2021); the change from 1991 to 2021 was then compared. To demonstrate the evolution of scientific production over time, graphs were constructed; data depicted were on the number and proportion of publications on NTDs in JBSTM and the NTDs with the highest proportion of scientific publications. Tables were organized by decades to present independent NTDs, types of scientific products, authors, descriptors, affiliations, and countries.

### Analysis of the bibliometric profile

Representative images of the relationships (maps) between authors, countries, institutions, and descriptors (nodes), the strength between these relationships (thickness of the arcs), and the number of their total contributions (node size), are presented. The “thesaurus” (VOSviewer tool) was applied to consolidate terms. The parameters considered were a maximum limit of “25” and a minimum of “2” for items of scientometric identification of each unit of analysis: 1) co-authorship by author; 2) co-authorship by countries; 3) co-authorship by organizations; and 4) co-occurrence of author keywords, linked to the bibliographic production relationship network and aggregated over the entire study period. For some parameters, we calculated the 95% confidence intervals (CI).

### Ethical aspects

The present study was based on *JBSTM* publications available on the 100% open access journal’s website (https://www.scielo.br/j/rsbmt/) free of charge. Thus, according to national guidelines, submission to a research ethics committee is not necessary. This study followed the ethical recommendations of the National Health Council (Resolution No. 466 of 2012).

## RESULTS

### General profile

**FIGURE 1:**
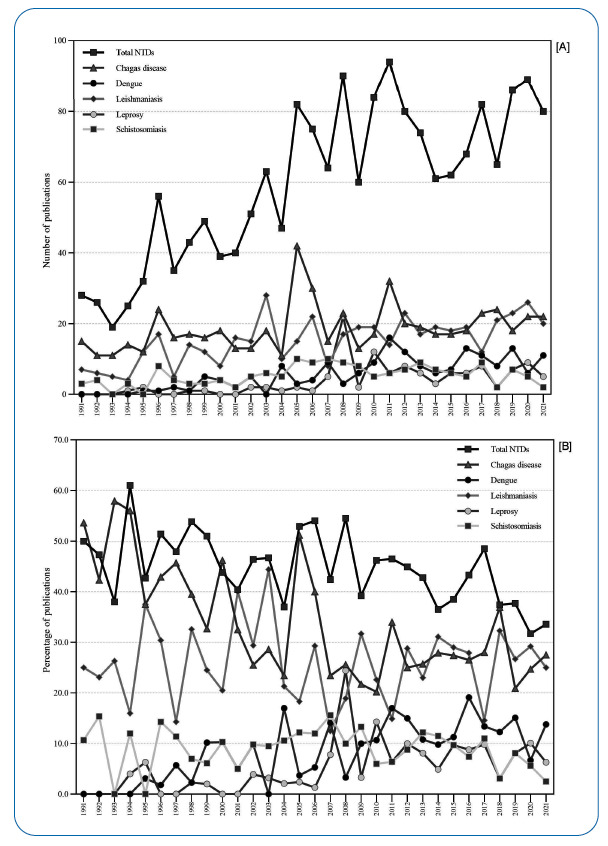
Number **[A]** and proportion **[B]** of publications on Neglected Tropical Diseases (Chagas disease, Dengue, Leishmaniasis, Leprosy, and Schistosomiasis) from the *Journal of the Brazilian Society of Tropical Medicine*, 1991-2021.

During the observation period of 31 years, *JBSTM* published a total of 4,268 scientific papers; of which, 1,849 (43.3%) were related to NTDs, which represented an annual average of 44.8% of all papers (CI 95%, 42.3-47.3) and a change of 52 publications (185.7%) from 1991 to 2021. The total number and percentage of publications increased over the period, with 17.0% (724/4,268) published during 1991-2000, 33.2% (1,416/4,268) from 2001-2010, and 49.9% (2,128/4,268) from 2011-2021. While the absolute number of NTDs publications increased about 2- to 4-fold, the proportion of NTDs publications reduced over time, from 48.6% (352/724) published during 1991-2000, to 46.3% (656/1,416) in 2001-2010, and 39.5% (841/2,128) in 2011-2021 (Table 2). There was a reduction in the relative number of publications made from 2017 onwards, with <40% occurring from 2018 to 2021. Some diseases also showed a reduction in the absolute number and percentage of publications (Figure 1A and Figure 1B). 

**TABLE 2:** Number, percentage, and changes of articles, organized by document and Neglected Tropical Diseases type published in the *Journal of the Brazilian Society of Tropical Medicine* during 1991-2000, 2001-2010, 2011-2021, and 1991-2021.

	Number of articles				Change
NTDs	1991-2000	2001-2010	2011-2020	1991-2021	1991 to 2021
	N (%)	N (%)	N (%)	N (%)	N (%)
**Total number of papers**	724 (17.0)	1,416 (33.2)	2,128 (49.9)	4,268 (100.0)	182 (325.0)
**Total number of NTD**s	352 (48.6)	656 (46.3)	841 (39.5)	1,849 (43.3)	52 (185.7)
**Document type**					
Article	296 (84.1)	597 (91.0)	723 (86.0)	1,616 (87.4)	40 (153.8)
Review	28 (8.0)	29 (4.4)	38 (4.5)	95 (5.1)	0 (-)
Conference Paper	11 (3.1)	10 (1.5)	0 (0.0)	21 (1.1)	-1 (-100.0)
Letter	10 (2.8)	12 (1.8)	26 (3.1)	48 (2.6)	4 (-)
Editorial	4 (1.1)	2 (0.3)	16 (1.9)	22 (1.2)	-1 (-100.0)
Note	2 (0.6)	4 (0.6)	32 (3.8)	38 (2.1)	9 (-)
Erratum	1 (0.3)	2 (0.3)	4 (0.5)	7 (0.4)	0 (-)
Short Communication	0 (0.0)	0 (00.0)	2 (0.2)	2 (0.1)	1 (-)
**NTDs**					
Chagas disease	154 (43.8)	195 (29.7)	232 (27.6)	581 (31.4)	7 (46.7)
Leishmaniasis	90 (25.6)	169 (25.8)	212 (25.2)	471 (25.5)	13 (185.7)
Dengue	14 (4.0)	49 (7.5)	111 (13.2)	174 (9.4)	11 (-)
Schistosomiasis	32 (9.1)	69 (10.5)	65 (7.7)	166 (9.0)	-1 (-33.3)
Leprosy	5 (1.4)	49 (7.5)	66 (7.8)	120 (6.5)	5 (-)
Paracoccidioidomycosis	18 (5.1)	28 (4.3)	26 (3.1)	72 (3.9)	0 (0.0)
Cryptococcosis	7 (2)	25 (3.8)	15 (1.8)	47 (2.5)	1 (-)
Rabies	5 (1.4)	16 (2.4)	22 (2.6)	43 (2.3)	3 (-)
Histoplasmosis	6 (1.7)	15 (2.3)	14 (1.7)	35 (1.9)	1 (-)
Chikungunya	0 (0.0)	0 (0.0)	30 (3.6)	30 (1.6)	2 (-)
Ascariasis	8 (2.3)	12 (1.8)	7 (0.8)	27 (1.5)	-1 (-100.0)
Taeniasis/cysticercosis	2 (0.6)	11 (1.7)	4 (0.5)	17 (0.9)	0 (-)
Sporotrichosis	2 (0.6)	4 (0.6)	10 (1.2)	16 (0.9)	0 (-)
Echinocococosis/Hydatidosis	0 (0.0)	3 (0.5)	11 (1.3)	14 (0.8)	3 (-)
Elephantiasis; Elephantiasis, Filarial	2 (0.6)	6 (0.9)	6 (0.7)	14 (0.8)	0 (-)
Trichuriasis	4 (1.1)	8 (1.2)	1 (0.1)	13 (0.7)	0 (-)
Snake Bites	2 (0.6)	7 (1.1)	4 (0.5)	13 (0.7)	1 (-)
Larva Migrans	3 (0.9)	5 (0.8)	4 (0.5)	12 (0.6)	0 (-)
Yaws	1 (0.3)	2 (0.3)	7 (0.8)	10 (0.5)	3 (-)
Trachoma	2 (0.6)	1 (0.2)	7 (0.8)	10 (0.5)	3 (-)
Scabies	3 (0.9)	3 (0.5)	2 (0.2)	8 (0.4)	1 (-)
Fascioliasis	0 (0)	1 (0.2)	6 (0.7)	7 (0.4)	0 (-)
Mycetoma	4 (1.1)	2 (0.3)	0 (0.0)	6 (0.3)	-1 (-100.0)
Myiasis	0 (0)	2 (0.3)	4 (0.5)	6 (0.3)	1 (-)
Chromomycosis	3 (0.9)	0 (0.0)	1 (0.1)	4 (0.2)	0 (-)
Onchocerciasis	4 (1.1)	0 (0.0)	0 (0.0)	4 (0.2)	0 (-)
Chromoblastomycosis	4 (1.1)	0 (0.0)	0 (0.0)	4 (0.2)	0 (-)
Coccidioidomycosis	1 (0.3)	1 (0.2)	1 (0.1)	3 (0.2)	0 (-)
Trypanosomiasis, African	1 (0.3)	0 (0.0)	1 (0.1)	2 (0.1)	0 (-)
Tungiasis	1 (0.3)	1 (0.2)	0 (0)	2 (0.1)	-1 (-)
Buruli ulcer	0 (0.0)	0 (0.0)	1 (0.1)	1 (0.1)	0 (-)
Ancylostomiasis	0 (0.0)	1 (0.2)	0 (0.0)	1 (0.1)	0 (-)
Opisthorchiasis	0 (0.0)	0 (0.0)	1 (0.1)	1 (0.1)	0 (-)
Lice Infestations	0 (0.0)	1 (0.2)	0 (0.0)	1 (0.1)	0 (-)
Dracunculiasis	0 (0.0)	0 (0.0)	0 (0.0)	0 (0.0)	0 (-)
Clonorchiasis	0 (0.0)	0 (0.0)	0 (0.0)	0 (0.0)	0 (-)
Paragonimiasis	0 (0.0)	0 (0.0)	0 (0.0)	0 (0.0)	0 (-)

**Key: -:** not calculated because of zero values; **NTDs:** Neglected Tropical Diseases; **JBSTM:** *Journal of the Brazilian Society of Tropical Medicine.*

Brazil appeared most frequently among authorships in the *JBSTM* publication, followed by other Latin American countries (Argentina and Mexico) during 1991-2000. There was a change in this profile during the 2001-2010 and 2011-2020 periods, with a greater number originating from the United States of America, Spain, and Belgium, among others (Table 3).

**TABLE 3:** Number of articles on Neglected Tropical Diseases in the *Journal of the Brazilian Society of Tropical Medicine*, according to the top ten authors, keywords, affiliation, and country, during 1991**-**2000, 2001**-**2010, 2011**-**2021, and 1991-2021.

1991-2000		2001-2010		2011-2020		1991-2021	
**Country**							
Brazil	288	Brazil	582	Brazil	752	Brazil	1,642
Argentina	7	United States	27	United States	30	United States	63
Mexico	4	Argentina	22	Colombia	20	Argentina	42
Portugal	4	Netherlands	9	Argentina	12	Colombia	21
Bolivia	3	Bolivia	7	Spain	10	Spain	14
Spain	3	Belgium	6	India	9	Bolivia	13
Venezuela	3	United Kingdom	5	Portugal	8	Mexico	13
Ecuador	2	Italy	4	Mexico	7	Portugal	13
Jordan	2	France	3	Peru	7	United Kingdom	13
China	1	Venezuela	3	United Kingdom	7	Netherlands	11
**Affiliations**							
Universidade de Brasília	35	Universidade Federal de Minas Gerais	89	Universidade de São Paulo	119	Fundação Oswaldo Cruz (Fiocruz) - Rio de Janeiro	201
Universidade Federal do Triângulo Mineiro	30	Fundação Oswaldo Cruz (Fiocruz) - Rio de Janeiro	84	Fundação Oswaldo Cruz (Fiocruz) - Rio de Janeiro	108	Universidade de São Paulo	199
Universidade de São Paulo	24	Universidade de São Paulo	56	Universidade Federal de Minas Gerais	75	Universidade Federal de Minas Gerais	182
Universidade Federal de Minas Gerais	18	Universidade Federal do Triângulo Mineiro	46	Centro de Pesquisa René Rachou (Fiocruz) - Minas Gerais	57	Universidade de Brasília	116
Universidade Federal do Maranhão	16	Centro de Pesquisa René Rachou (Fiocruz) - Minas Gerais	41	Universidade Federal do Ceará	45	Centro de Pesquisa René Rachou (Fiocruz) - Minas Gerais	112
Centro de Pesquisa René Rachou (Fiocruz) - Minas Gerais	14	Universidade de Brasília	38	Universidade de Brasília	43	Universidade Federal do Triângulo Mineiro	104
Centro de Pesquisas Gonçalo Moniz (Fiocruz) - Bahia	10	Universidade Federal de Goiás	30	Universidade Estadual Paulista	42	Universidade Federal do Ceará	63
Fundação Oswaldo Cruz (Fiocruz) - Rio de Janeiro	9	Centro de Pesquisas Aggeu Magalhães (Fiocruz) - Pernambuco	22	Centro de Pesquisas Aggeu Magalhães (Fiocruz) - Pernambuco	32	Centro de Pesquisas Aggeu Magalhães (Fiocruz) - Pernambuco	59
Universidade Federal de Uberlândia	9	Universidade Federal de Pernambuco	22	Fundação de Medicina Tropical Doutor Heitor Vieira Dourado (Amazonas)	28	Universidade Estadual Paulista	55
Superintendência de Controle de Endemias - São Paulo (SP)	7	Universidade Federal do Rio de Janeiro	21	Universidade Federal do Triângulo Mineiro	28	Universidade Federal de Pernambuco	52
**Authors**							
Marsden P.D.	22	Lambertucci J.R.	39	Camargo L.M.A.	18	Lambertucci J.R.	54
Amato Neto V.	15	Prata A.	20	Rosa J.A.	16	Prata A.	40
Costa J.M.	15	Bührer-Sékula S.	13	Guerra J.A.O.	15	Correia D.	24
Lopes E.R.	13	Carlier Y.	12	Meneguetti D.U.O.	15	Dias J.C.P.	22
Prata A.	13	Torrico F.	11	Dias J.C.P.	13	Marsden P.D.	22
Chapadeiro E.	11	Coura J.R.	10	Dias E.S.	12	Coura J.R.	21
Carvalho E.M.	9	Voieta I.	10	Correia D.	11	Rosa J.A.	19
Amato V.S.	8	Borges-Pereira J.	9	Lambertucci J.R.	11	Silva A.R.	19
Castro C.	7	Severo L.C.	9	Oliveira J.	10	Camargo L.M.A.	18
Rocha A.	7	Dias J.C.P.	9	Figueiredo L.T.M.	9	Castro C.	18
**Keywords**							
Leishmaniasis	19	Leishmaniasis	103	Leishmaniasis	160	Leishmaniasis	290
Chagas Disease	12	Chagas disease	94	Chagas disease	137	Chagas disease	245
Treatment	6	Epidemiology	55	Epidemiology	79	Epidemiology	140
Control	4	Leprosy	41	Leprosy	51	Leprosy	95
HIV/Aids	4	Schistosomiasis	39	Triatomine	43	Schistosomiasis	90
*Trypanosoma cruzi*	4	*Trypanosoma cruzi*	38	*Trypanosoma cruzi*	41	*Trypanosoma cruzi*	87
Mefloquine	3	Dengue	36	Diagnosis	36	Dengue	85
Pentavalent Antimonials	3	Control	16	Dengue	35	Triatomine	54
Sodium Stibogluconate	3	Paracoccidioidomycosis	15	Schistosomiasis	29	Diagnosis	52
Specific Treatment	3	Serology	15	Chikungunya	27	HIV/Aids	39

Data extracted on 07-27-2022.

The Oswaldo Cruz Foundation (Fiocruz) (From Rio de Janeiro) was among the affiliated institutions of authors with the greatest number of publications, followed by the University of São Paulo (USP) and the Federal University of Minas Gerais (UFMG). The University of Brasilia (UnB) presented the highest number of bibliographic productions of NTDs from 1991 to 2000, ranking sixth during 2001-2010 and 2011-2021. The Federal University of Ceará, in the period from 2011 to 2021, occupied the fifth (45/841) and seventh positions (63/1,849) (Table 3).

Original articles represented the most commonly published type of document, with more than 87.4% (1991-2000: n=296, 84.1%; 2001-2010: n=597, 91.0%; 2011-2021: n=723, 86.0%) of total publications, followed by reviews. Other publication types (‘letters’, ‘editorials’, and ‘notes’) accounted for less than 8% of the total number of publications (Table 2).

The highest proportion of publications were related to Chagas disease (31.4%, 581/1,849), leishmaniasis (25.5%, 471/1,849), dengue (9.4%, 174/1,849), schistosomiasis (9.0%, 166/1,849), and leprosy (6.5%, 120/1,849). Three NTDs did not appear in any publications in the *JBSTM* throughout the study period: dracunculiasis, clonorchiasis, and paragonimiasis (Table 2).

Chagas disease stood out for the period, with an average by-year proportion of 33.9% (95% CI, 29.9-37.9) for publications related to NTDs (in terms of number and percentage). Among the five NTDs with the highest number of publications in the *JBSTM*, only publications on dengue showed an increasing pattern over the analyzed period (Figure 1A and Figure 1B).

### Bibliometric profile

The most frequently appearing authors were “*Marsden, P.D.*” (22/352), “*Amato Neto V.*” (15/352), and “*Costa, J.M.*” (15/352), during the 1991-2000 period; “*Lambertucci, J.R.*” (39/656) and “*Prata, A.*” (20/656) during 2001-2010; “*Camargo, L.M.A.*” (18/841) and “*Rosa J.A.*” (16/841) during 2011-2021; and “*Lambertucci J.R.*” (54/1,849) and “*Prata A.*” (40/1,849) for the overall total across all periods (Table 3). The descriptors “*leishmaniasis*” (1991-2000: 19/352, 2001-2010: 103/656, 2011-2021: 160/841; total: 290/1,849) and *“Chagas disease*” (1991-2000: 12/352, 2001-2010: 94/656, 2011-2021: 137/841; total: 245/1,849) were the most used throughout the three analysis periods. For the 2001-2010 and 2011-2021 periods, the terms “*epidemiology*” (2001-2010: 55/656, 2011-2021: 79/761) and “*leprosy*” (2001-2010: 41/656, 2011-2021: 51/761) appeared as the 3rd and 4th most cited terms, respectively (Table 3).

**FIGURE 2:**
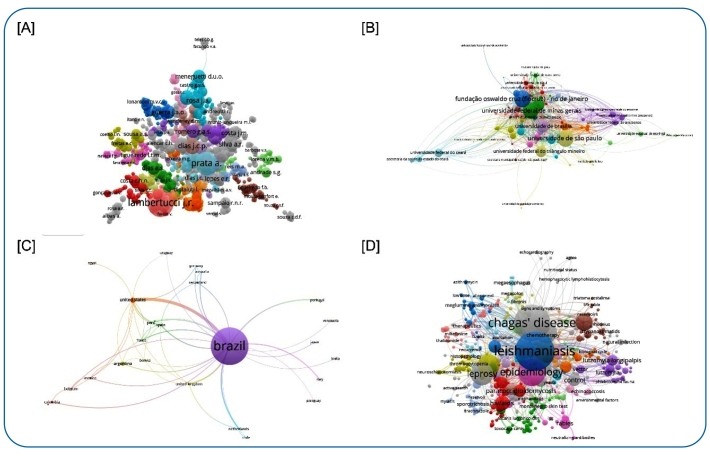
List of bibliographic production on Neglected Tropical Diseases from 1991-2021 in the *Journal of the Brazilian Society of Tropical Medicine*, according to **[A]** authors, **[B]** organizations, **[C]** countries, and **[D]** keywords.

For co-authorship of author relationships, from 1991-2021, 1,306 authors with more than two documents were identified and organized into 33 clusters, and the eight most productive clusters showed more than 37 correlated authors. The most prominent authors with articulated connections are “*Prata A.*” and “*Lambertucci J.R.*”. (Figure 2A).

The co-authorship and organization relationship presented a pattern among the institutions for the 1991-2021 period, with 167 presenting more than two publications, and was organized into 16 clusters. The leading institution for scientific production was the *“Fundação Oswaldo Cruz (Fiocruz) - Rio de Janeiro*”, followed by the “*Universidade de São Paulo*”. Notable volumes were also completed by the “*Universidade Federal de Minas Gerais*”, “*Universidade de Brasília*”, “*Centro de Pesquisa René Rachou (Fiocruz) - Minas Gerais*”, “*Universidade Federal do Triângulo Mineiro*”, and “*Universidade Federal do Ceará*” (Figure 2B).

The analysis of co-authorship across countries showed a predominance of Brazilian connections to other countries. We identified 34 countries in which authors presented two or more publications. The strongest cooperation links were observed between Brazil and the United States, Argentina, and the United Kingdom (Figure 2C).

An analysis of the co-occurrence of author keywords for the 1991-2021 period identified 467 descriptors, with two or more citations, and was organized into 36 clusters. The most prominent cluster is “*leishmaniasis*,” followed by “*Chagas disease*”. Other secondary clusters are also noteworthy, such as “*schistosomiasis*,” “*leprosy*,” “*Trypanosoma cruzi*”, and “*epidemiology*” (Figure 2D). During this period, most of the published articles were in the English language (62.5%; 2669/4268).

## DISCUSSION

We found that the vast majority of papers were published by Brazilian authors, and whether a disease was endemic or not to Brazil did not play a role in the published studies. This is not a surprise, as among the diseases defined as NTDs by the WHO, some are not considered endemic or do not occur in Brazil. The *JBSTM*, through its traditions, national reach, and interdisciplinary openness, tends to aggregate Brazilian researchers and expand on collaborations with other countries; this is due to a need to prioritize the journal’s theme, progressive increase in international visibility, and its impact factor, to aid in the achievement of of the Millennium Development Goals (MDGs) formulated in the years 2000^13^. There were additional scientific productions in the *JBSTM* linked to institutions in the South and Southeast of Brazil, likely due to the distribution of teaching and research institutions in Tropical Medicine, and the differential funding available from governments and funding agencies. This difference may be due to tradition, better environment, and infrastructure. Given the endemic contexts of Brazil and Latin America, it is important to recognize the outstanding number of publications on Chagas disease, leishmaniasis, schistosomiasis, dengue, and leprosy; these account for most of the publications on NTDs in the *JBSTM* in human, parasitological, and entomological research. There are other factors contributing to the greater number of Brazilian authors published in the *JBSTM*, such as the inclusion of articles written in Portuguese until 2012 and the tendency of the *BSTM* to publish their work in this journal.

Since *JBSTM* publishes articles on research conducted in Brazil and Latin America^12^, diseases with more publications reflects those which are a priority for Brazil and are present in the list of compulsory notifications^14^. NTDs with the highest percentage of scientific production in the *JBSTM* (with an increase or decrease in the absolute number and percentage over time) are endemic in Brazil, with a considerable number of cases recorded every year, or even in epidemic years, such as dengue^7^ ^,^ ^9^ ^,^ ^10^.

In recent decades, there have been constant changes to the world demography, which may influence the increases in infectious diseases (especially NTDs): high population growth, acceleration of urbanization, increased life expectancy, decreased fertility rates, and population aging. These changes have influenced the unplanned growth of cities associated with poverty, unemployment, inadequate housing, agglomerations, increased vector-borne diseases, increased vehicle traffic, environmental degradation, and pollution. Such variations also represent changes in the behavior of different agents that cause infectious diseases^15^.

National and international agendas highlighting NTDs from the year 2000^13^ did not increase the number and/or percentage of publications in the *JBSTM*, either in general or in specific productions, as demonstrated in other studies^8^ ^,^ ^16^ ^,^ ^17^. This change may also be related to evolutions in the pattern of research, with the search for journals with higher ratings primarily according to the impact factor occurring more frequently, as it was a requirement of research funding agencies. NTDs are included in research calls for proposals from funding agencies such as CNPq (portuguese: *Conselho Nacional de Desenvolvimento Científico e Tecnológico*), FAPESP (portuguese: *Fundação de Amparo à Pesquisa do Estado de São Paulo*), FAPERJ (portuguese: *Fundação de Amparo à Pesquisa do Estado do Rio de Janeiro*), and FAPEMIG (portuguese: *Fundação de Amparo à Pesquisa do Estado de Minas Gerais*)^8^. *JBSTM* has a growing impact factor, going from 1.161 in 2016 to 2.141 in 2021, occupying the fourth position in Brazilian journals and tenth worldwide in the area of Tropical Medicine, with 100% open access and an expressive volume of citations. In the Brazilian context, the “*Qualis Periódicos*” of CAPES has also regulated the standards of publications related to postgraduate programs in the country, which has brought significant impacts, such as driving leading researchers and postgraduate students to seek journals with better ranking^18^.

*JBSTM* has many publications on endemic NTDs in Brazil^9^. This pattern differs from that found in research from other Latin American countries, where dengue usually has the highest average proportion of studies, followed by leishmaniasis, Chagas disease, and schistosomiasis^19^. The fact that *JBSTM* represents the Brazilian context from a greater perspective means that some diseases stand out as containing a greater number of publications, particularly because they are public health problems and are highlighted in government agendas^7^ ^,^ ^9^.

Articles and reviews were among the most common publication types accepted by *JBSTM*, similar to those found in other studies using a general approach from various journals^19^ ^,^ ^20^. The two most commonly accepted formats are based on the traditional formats of NTDs articles published in scientific journals^7^.

The scientific production of NTDs in *JBSTM* is highlighted among authors when considering references for their work with specific diseases, such as schistosomiasis^21^, Chagas disease^22^ ^,^ ^23^, and leishmaniasis^24^. The most cited authors, besides being references in their areas of expertise, also contributed to the consolidation of public health policies, such as chronic Chagas disease, which became a compulsory notification in May 2020^22^ ^,^ ^23^ ^,^ ^25^.

The most commonly mentioned descriptors for the retrieval of scientific productions on NTDs from *JBSTM* revealed a strategic pattern of publications in the journal, a fact not observed in other scientific journals that publish on diseases with more general endemic characteristics in Latin America, Asia, and other continents^19^ ^,^ ^26^ ^,^ ^27^. The descriptors express the general aspects recommended by government agencies, with priority for recurrent diseases in the Brazilian scenario, and consequently, the area of expertise of the *JBSTM* authors^7^ ^,^ ^9^ ^,^ ^22^. Climate change scenarios may contribute to the emergence and/or re-emergence of some NTDs, especially those transmitted by vectors or intermediate hosts; consequently, research published in *JBSTM* could contribute to surveillance and monitoring^28^.

Some institutions have a higher proportion of publications in *JBSTM*, mainly those from the states of Rio de Janeiro, Minas Gerais, and São Paulo, with an increased number from the northeast region: *Centro de Pesquisas Aggeu Magalhães* (Fiocruz Pernambuco), *Universidade Federal de Pernambuco*, and *Universidade Federal do Ceará*; and in the North region: *Fundação de Medicina Tropical Doutor Heitor Vieira Dourado* (Amazonas). This pattern is not significantly different from the international pattern of other journals that publish on NTDs and institutions, where these institutions are also hegemonic^16^ ^,^ ^29^. This difference occurs mainly in the funding that these research institutions receive from the development agencies^30^. However, research could be directed towards the presentation of strategies for the control and elimination of NTDs present in the national scenario and Latin America, which will be even more relevant in the pandemic context and for the future post-pandemic, with the risk of even more limited resources for the resumption of public health programs in the coming years^31^.

Due to the Brazilian context, the majority of *JBSTM* publications are concentrated from Brazil; however, affiliations in institutions from other countries in Latin America, North America, Europe, and Asia have publications and connections with Brazilian authors. This is similar to the results presented in a study that evaluated publications in NTDs in Latin America and the Caribbean^19^. The co-authorship connections between scientific production in the United States of America increased after 2000, with the prioritization of NTDs in international agendas^2^ ^,^ ^13^. Other studies have also shown Brazil's connection to other countries^16^ ^,^ ^32^.

This study presents with limitations, such as the use of a single database (Scopus®). In addition, Scopus® groups the publications from "Images in Infectious Diseases" or "Case Reports" in the articles. the search based on the selection of scientific production from terms in the titles, abstracts, and keywords for greater sensitivity; the affiliation between countries of the authors, facts that may not translate the reality due to the quality of the filling out that, in some cases, it is not possible to recognize the institution; the absence of analysis of the citation profile of the manuscripts approaching different diseases analyzed. Despite such limitations, the findings of the current study are justified and contribute to the evaluation of the current scenario of scientific production of NTDs in one of the traditional journals which is also most relevant to Brazilian Tropical Medicine.

## CONCLUSION

*JBSTM* remains an important vehicle for disseminating research on NTDs during this period despite the proportional reduction seen in recent years. The disparity between the institutions that work and publish NTDs in the country is evident. There is a need to strengthen the research and subsequent publications on NTDs. Institutions working and publishing on NTDs in the country were concentrated in the South and Southeast regions, showing that additional investments in institutions in other regions of the country are required. The NTDs-related publications of *JBSTM* detail the current challenges faced by the Brazilian Unified Health System (in portuguese: *Sistema Único de Saúde* [SUS]).
